# Supplementary material for: A Cost–Consequence Analysis of Nutritional Interventions Used in Hospital Settings for Older Adults with or at Risk of Malnutrition
Source: Healthcare (Basel). 2024 May 17;12(10):1041. doi: 10.3390/healthcare12101041 (PMC11120964; doi:10.3390/healthcare12101041)
Supplement: Supplementary file 1 [file healthcare-12-01041-s001.zip › healthcare-2966459-supplementary.pdf]

Supplementary Material

Supplementary Table S1. Cost of Resources Utilized.

Resource Item	Unit	Cost (US$)			Notes	Source of Cost
		Lower	Median	Upper		
Allied Health Professional (AHP) Initial or Follow Up Visit (Inpatient)	Per Minute	$0.49	$0.63	$0.81	# Remuneration of AHPs in Singapore Public Hospitals follows a similar pay rate across the different professions. Senior AHP grade	Hospital
Nursing Initial Visit (Nutrition Support Team) (Inpatient)	Per Minute	$0.49	$0.63	$0.81	^ Senior Registered Nurse or Staff Nurse grade	Hospital
Doctor Visit (Nutrition Support Team) (Inpatient)	Per Minute	$0.91	$1.02	$1.36	* Associate Consultant / Consultant	Hospital
Nurse Assistant/ / Healthcare Assistant/ Diet or Therapy Assistant/ / Coordinators (Inpatient)	Per Minute	$0.08	$0.16	$0.24	** Enrolled Nurse / Healthcare Asst / Therapist Asst / Dietetic Asst / Coordinator	Hospital
Dietitian follow up Visit (Outpatient/ Home/ Community)	Per Minute	$0.49	$0.63	$0.81	Refer to #	Hospital
Nursing Follow-up Visit (Nutrition Support Team) (Outpatient/ Home/ Community)	Per Minute	$0.49	$0.63	$0.81	Refer to ^	Hospital
Doctor Follow-up Visit (Nutrition Support Team) (Outpatient/ Home/ Community)	Per Minute	$0.91	$1.02	$1.36	Refer to *	Hospital
General Practitioner Visit (Private Home / House-call)	Per Visit	$140.71	$156.34	$195.42	Visit from Private GPs outside the hospital settings (non-emergency)	https://www.homage.sg/services/house-call-doctor/ https://speedoc.com/sg/house-call-doctors
General Practitioner Visit (Private Clinic Walk-in)	Per Visit	$15.63	$23.45	$42.99	Visit to Private GPs outside the hospital settings	https://singapore-medical.com/medical-costs
General Practitioner Visit (Polyclinics - Subsidised)	Per Visit	$5.39	$10.94	$21.65	Visit to Public GPs in Subsidised Govt Polyclinics	https://polyclinic.singhealth.com.sg/patient-care/charges-payment https://www.nup.com.sg/Pages/Fees%20and%20Charges/queenstownp.aspx https://www.nhgp.com.sg/charges_and_payment/
Nurse Assistant/ / Healthcare Assistant/ / Diet or Therapy Assistant/ / Coordinators (Outpatient/ Home/ Community)	Per Minute	$0.08	$0.16	$0.24	Refer to **	Hospital
Exercise instructor / Trainers / Assistant/ Trainers (Outpatient/ Home/ Community)	Per Minute	$0.27	$0.34	$0.42	Refer to ^^	Hospital

Resource Item	Unit	Cost (US$)			Notes	Source of Cost
		Lower	Median	Upper		
Staff travel - Allied Health Professional (AHP)	Per Minute	$0.41	$0.61	$0.81	Determined by time spent on traveling, based on per minute pay rate of a Senior AHP listed in #, excluding transport allowances. Assuming a maximum of 10km radius coverage from the hospital location.	Hospital
Staff travel– Nursing	Per Minute	$0.27	$0.34	$0.42	Determined by time spent on traveling, based on per minute pay rate of a Senior Nurse listed in ^, excluding transport allowances. Assuming a maximum of 10km radius coverage from the hospital location.	Hospital
Staff travel– Doctor	Per Minute	$0.91	$1.02	$1.36	Determined by time spent on traveling, based on per minute pay rate of a medical doctor listed in **, excluding transport allowances. Assuming a maximum of 10km radius coverage from the hospital location.	Hospital
Staff travel – Exercise instructor / Trainers / Asst Trainers	Per Minute	$0.20	$0.28	$0.41	Determined by time spent on traveling, based on per minute pay rate of an Exercise instructor / Trainers / Asst Trainers listed in ^^, excluding transport allowances. Assuming a maximum of 10km radius coverage from the hospital location.	Hospital
Staff travel - Nurse Asst / Healthcare Asst/ Diet or Therapy Asst / Coordinators	Per Minute	$0.08	$0.16	$0.24	Determined by time spent on traveling, based on per minute pay rate of a Nurse Asst / Healthcare Asst/ Diet or Therapy Asst / Coordinators listed in **, excluding transport allowances. Assuming a maximum of 10km radius coverage from the hospital location.	Hospital
Food for Special Medical Purposes - Oral nutritional supplements	Per Serve	$1.95	$2.74	$4.69	Standard 1.0kcal to High Protein/Kcal ONS	Wong A, Sowa PM, Banks MD, Bauer JD. Home Enteral Nutrition in Singapore's Long-Term Care Homes-Incidence, Prevalence, Cost, and Staffing. Nutrients. 2019;11(10).
Food for Special Medical Purposes - Protein powder supplement	Per Serve	$0.34	$0.36	$0.38	Standard whey protein powder	Wong A, Sowa PM, Banks MD, Bauer JD. Home Enteral Nutrition in Singapore's Long-Term Care Homes-Incidence, Prevalence, Cost, and Staffing. Nutrients. 2019;11(10).
Vitamins	Per Serve (tablet)	$0.08	$0.16	$0.23	Standard Generic Multivitamin (Tablet)	Retail, Hospital Pharmacy https://www.lazada.sg/shop/pharmacare/?path=index.htm
Vitamin D	Per Serve (tablet)	$0.03	$0.05	$0.08	Standard Generic Soft Gel Vitamin D3 (Capsule)	Retail, Hospital Pharmacy https://www.lazada.sg/shop/pharmacare/?path=index.htm
Minerals or Trace Elements	Per Serve (tablet)	$0.08	$0.16	$0.23	Standard Generic Mineral/Trace Elements (Tablet)	Retail, Hospital Pharmacy https://www.lazada.sg/shop/pharmacare/?path=index.htm

Resource Item	Unit	Cost (US$)			Notes	Source of Cost
		Lower	Median	Upper		
Calcium	Per Serve (tablet)	$0.10	$0.16	$0.23	Standard General Calcium Carbonate or Calcium Acetate	Retail, Hospital Pharmacy https://www.lazada.sg/shop/pharmacare/?path=index.htm
Educational Pamphlets and Resources	Per Page	$0.08	$0.09	$0.11	Printing services engaged by hospital (based on lowest value quotations)	Hospital
Volunteer Visit	Per Minute	$0.00	$0.00	$0.00	Free, Volunteers sourced from Hospital Volunteer Services	Free
Hospital Parking Waiver for Volunteer	Per Day	$11.02	$11.02	$11.02	Hospital Carpark Season Parking	Hospital
Laboratory Tests - Basic	Per Test	$10.94	$17.20	$23.45	Hospital Standard Laboratory Tests for Renal Panel, LFTs and FBC	Hospital
Phone Consults / Telehealth - AHPs	Per Minute	$0.41	$0.61	$0.81	Determined by time spent on phone consult or telehealth, excluding technology implementation cost and land line phone bills	Hospital
Phone Consults / Telehealth - Nurse	Per Minute	$0.27	$0.34	$0.42	Determined by time spent on phone consult or telehealth, excluding technology implementation cost and land line phone bills	Hospital
Phone Consults/ Telehealth - Exercise instructor / Trainers / Assistant Trainers	Per Minute	$0.20	$0.28	$0.41	Determined by time spent on phone consult or telehealth, excluding technology implementation cost and land line phone bills	Hospital
Corporate Landline Call Charges (Peak Hour)	Per Minute	$0.02	$0.02	$0.02	Corporate Land Line subscription	Retail, SingTel
Supplement Delivery	Per Trip	$11.73	$11.73	$11.73	based on one trip per month fee of $15, quotation from hospital pharmacy	Hospital
Exercise Weights	Per Item	$3.91	$7.82	$19.54	Range from 1kg to 5 kg weights	Retail https://www.decathlon.sg/
Resistive Band	Per Item	$2.35	$3.91	$10.94	Range from 1kg to 5kg resistive bands	Retail https://www.decathlon.sg/
Special meals in hospital	Per Serve	$1.56	$3.91	$6.25	Non-standard meals catered from hospital kitchen	Hospital
Additional Snack	Per Item	$0.39	$1.56	$3.91	Dependent of type of snack, e.g. jelly, pudding, high protein sandwiches and cakes available from hospital food services	Retail
High Protein Yoghurt	Per Item	$1.17	$1.56	$2.35	Containing minimally 10g protein per serve	Retail
High Protein Bread	Per Item	$0.23	$0.47	$0.78	Containing minimally 3g protein per serve	Retail
Learner Packs for Trainees	Per Item	$1.56	$3.91	$7.82	Learning packs that contained items such as hospital food charts, examples of menus and handouts for trainees / participants	Retail
Training by AHPs to Implement Intervention	Per Minute	$0.41	$0.61	$0.81	Refer to #	Hospital
Training by Nurse to Implement Intervention	Per Minute	$0.27	$0.34	$0.42	Refer to ^	Hospital

Resource Item	Unit	Cost (US$)			Notes	Source of Cost
		Lower	Median	Upper		
Training attended by Nurse Asst / Healthcare Asst/ Diet Asst / Coordinators	Per Minute	$0.08	$0.16	$0.24	Refer to **	Hospital
Training attended by Nursing	Per Minute	$0.27	$0.34	$0.42	Refer to ^	Hospital
Equipment – Hand Grip Dynamometer	Per Set	$769.19	$1,133.46	$1,547.76	1 set required for intervention	Retail https://rehabmart.com.sg/physiotherapy-rehab-therapy/evaluation-assessment/hand-dynamometer-plus-digital-jamar-fb120604
Equipment – Skinfold Callipers	Per Set	$448.69	$496.38	$687.89	1 set required for intervention	Retail https://www.amazon.sg/s?k=harpenden+skinfold+caliper&sprefix=harpenden%2Caps%2C433&ref=nb_sb_ss_ts-doa-p_1_9
Height measuring gauge) / Stadiometer	Per Set	NA	NA	NA	Available in All Public Hospitals' Clinics - Stadiometer and Height Measuring Gauge.	
Hip protectors	Per Set	$22.67	$39.08	$120.38	1 set per patient required for intervention if necessary	https://rehabshop.com.sg/hip-protector https://www.ninelife.sg/products/meditex-hip-protector-xl
Bio-impedance Analysis	Per Test	$15.63	$23.45	$39.08	1 set required for intervention	https://www.amazon.sg/s?k=bio+impedance+analysis+scale&crid=9LGCGIHPNHSU&sprefix=bio+impedance+analysis+scale%2Caps%2C423&ref=nb_sb_noss
Equipment – Nutrition Analysis Software	Subscription per month	$25.80	$49.25	$97.71	1 set required for intervention	Retail
Assistance with installation of assistive devices	Per Installation	$0.00	$39.08	$78.17	As required for each patient Free if using Senior Mobilty Enabling Fund	https://www.aic.sg/financial-assistance/seniors-mobility-enabling-fund

Study or Subgroup	NS (No EN PN) Events	NS (No EN PN) Total	Control Events	Control Total	Weight	Risk Ratio M-H, Random, 95% CI
**18.2.1 <1 month**						
Vlaming et al, 2001	12	275	14	274	1.6%	0.85 [0.40, 1.81]
Vermeeren 2004	0	29	0	27		Not estimable
Stelten 2015	0	28	0	28		Not estimable
Starke 2011	11	66	11	66	1.6%	1.00 [0.47, 2.14]
Soderstrom 2020	21	168	9	54	1.8%	0.75 [0.37, 1.54]
Saudny-Unterberger 1997	1	17	1	16	0.1%	0.94 [0.06, 13.82]
Rana 1992	0	20	0	20		Not estimable
Potter 2001	21	186	33	195	3.5%	0.67 [0.40, 1.11]
Olofsson 2007	9	102	13	97	1.4%	0.66 [0.29, 1.47]
Niccoli 2017	0	27	0	26		Not estimable
Myint 2013	1	61	1	60	0.1%	0.98 [0.06, 15.37]
Munk 2014	1	42	1	38	0.1%	0.90 [0.06, 13.97]
McEvoy 1982	0	26	0	25		Not estimable
MacFie 2000	4	75	1	25	0.2%	1.33 [0.16, 11.38]
Keele 1997	2	47	0	53	0.1%	5.63 [0.28, 114.27]
Kaegi-Braun 2020	231	994	246	999	25.1%	0.94 [0.81, 1.10]
Johansen 2004	9	108	6	104	0.9%	1.44 [0.53, 3.92]
Houwing 2003	0	51	0	52		Not estimable
Holyday 2012	4	71	1	72	0.2%	4.06 [0.46, 35.41]
Hogarth 1996	5	55	8	51	0.9%	0.58 [0.20, 1.66]
Hoekstra 2011	1	61	2	66	0.2%	0.54 [0.05, 5.82]
Hickson 2004	31	292	35	300	4.3%	0.91 [0.58, 1.44]
Gazzotti 2003	2	39	2	41	0.3%	1.05 [0.16, 7.10]
Gariballa 2006	32	223	19	222	3.2%	1.68 [0.98, 2.87]
Faxen-Irving 2011	0	34	2	37	0.1%	0.22 [0.01, 4.37]
Ekinci 2016	0	38	0	37		Not estimable
Dogliotto 1996	66	338	71	340	9.2%	0.94 [0.69, 1.26]
de Sousa 2012	0	20	2	17	0.1%	0.17 [0.01, 3.34]
Cano-Torres 2017	1	28	5	27	0.2%	0.19 [0.02, 1.55]
Botella-Carretero 2008	0	60	0	30		Not estimable
Beelen 2018	1	77	0	82	0.1%	3.19 [0.13, 77.20]
Arias 2008	46	262	31	267	5.0%	1.51 [0.99, 2.31]
Anbar 2014	0	22	2	28	0.1%	0.25 [0.01, 5.00]
Subtotal (95% CI)		3942		3776	60.5%	0.96 [0.86, 1.08]
Total events	512		516			
Heterogeneity: $Tau^2 = 0.00$; $Chi^2 = 22.81$, df = 24 (P = 0.53); $I^2 = 0\%$						
Test for overall effect: Z = 0.65 (P = 0.52)						
**18.2.2 1-3 months**						
Miller 2006	1	24	0	26	0.1%	3.24 [0.14, 75.91]
Malafarina 2017	5	55	1	13	0.2%	1.18 [0.15, 9.27]
Gariballa 1998	2	20	7	20	0.5%	0.29 [0.07, 1.21]
Espaulella 2000	17	85	10	86	1.8%	1.72 [0.84, 3.54]
Duncan 2006	19	145	36	157	3.5%	0.57 [0.34, 0.95]
Delmi 1990	6	27	10	32	1.2%	0.71 [0.30, 1.70]
Broqvist 1994	1	9	1	13	0.1%	1.44 [0.10, 20.21]
Beck 2015	2	34	6	37	0.4%	0.36 [0.08, 1.68]
Banerjee 1978	4	31	6	32	0.7%	0.69 [0.21, 2.21]
Abalan 1992	0	15	0	14		Not estimable
Subtotal (95% CI)		445		430	8.5%	0.76 [0.50, 1.15]
Total events	57		77			
Heterogeneity: $Tau^2 = 0.08$; $Chi^2 = 10.05$, df = 8 (P = 0.26); $I^2 = 20\%$						
Test for overall effect: Z = 1.30 (P = 0.19)						
**18.2.3 >3 months**						
Yoshimura 2016	0	19	0	17		Not estimable
Yang 2019	9	39	13	43	1.7%	0.76 [0.37, 1.59]
Wyers 2018	6	73	5	79	0.7%	1.30 [0.41, 4.07]
Volkert et al, 1996	4	35	8	37	0.8%	0.53 [0.17, 1.60]
Vázquez-Sánchez 2019	6	52	6	54	0.8%	1.04 [0.36, 3.01]
Terp 2018	12	67	10	67	1.6%	1.20 [0.56, 2.59]
Soderstrom 2020	48	339	16	110	3.3%	0.97 [0.58, 1.64]
Singh 2012	4	62	8	62	0.7%	0.50 [0.16, 1.58]
Shyu 2013	8	99	6	99	0.9%	1.33 [0.48, 3.70]
Sharma 2017	23	78	22	70	3.8%	0.94 [0.58, 1.53]
Rondanelli 2016	0	69	0	61		Not estimable
Persson 2007	6	51	12	57	1.1%	0.56 [0.23, 1.38]
Neelemaat 2012	20	104	22	104	3.1%	0.91 [0.53, 1.56]
Milte 2016	4	86	4	89	0.5%	1.03 [0.27, 4.01]
Larsson 1990	29	197	55	238	5.3%	0.64 [0.42, 0.96]
Feldblum 2011	3	78	21	181	0.7%	0.33 [0.10, 1.08]
Deutz 2016	15	313	30	309	2.6%	0.49 [0.27, 0.90]
Casals 2015	6	52	6	53	0.8%	1.02 [0.35, 2.96]
Bonilla-Palomas 2016	9	59	18	61	1.8%	0.52 [0.25, 1.06]
Beck 2012	4	73	7	79	0.7%	0.62 [0.19, 2.03]
Subtotal (95% CI)		1945		1870	31.0%	0.76 [0.64, 0.91]
Total events	216		269			
Heterogeneity: $Tau^2 = 0.00$; $Chi^2 = 13.45$, df = 17 (P = 0.71); $I^2 = 0\%$						
Test for overall effect: Z = 3.10 (P = 0.002)						
**Total (95% CI)**		6332		6076	100.0%	0.88 [0.80, 0.97]
Total events	785		862			
Heterogeneity: $Tau^2 = 0.00$; $Chi^2 = 52.44$, df = 51 (P = 0.42); $I^2 = 3\%$						
Test for overall effect: Z = 2.59 (P = 0.010)						
Test for subgroup differences: $Chi^2 = 5.65$, df = 2 (P = 0.06), $I^2 = 64.6\%$						

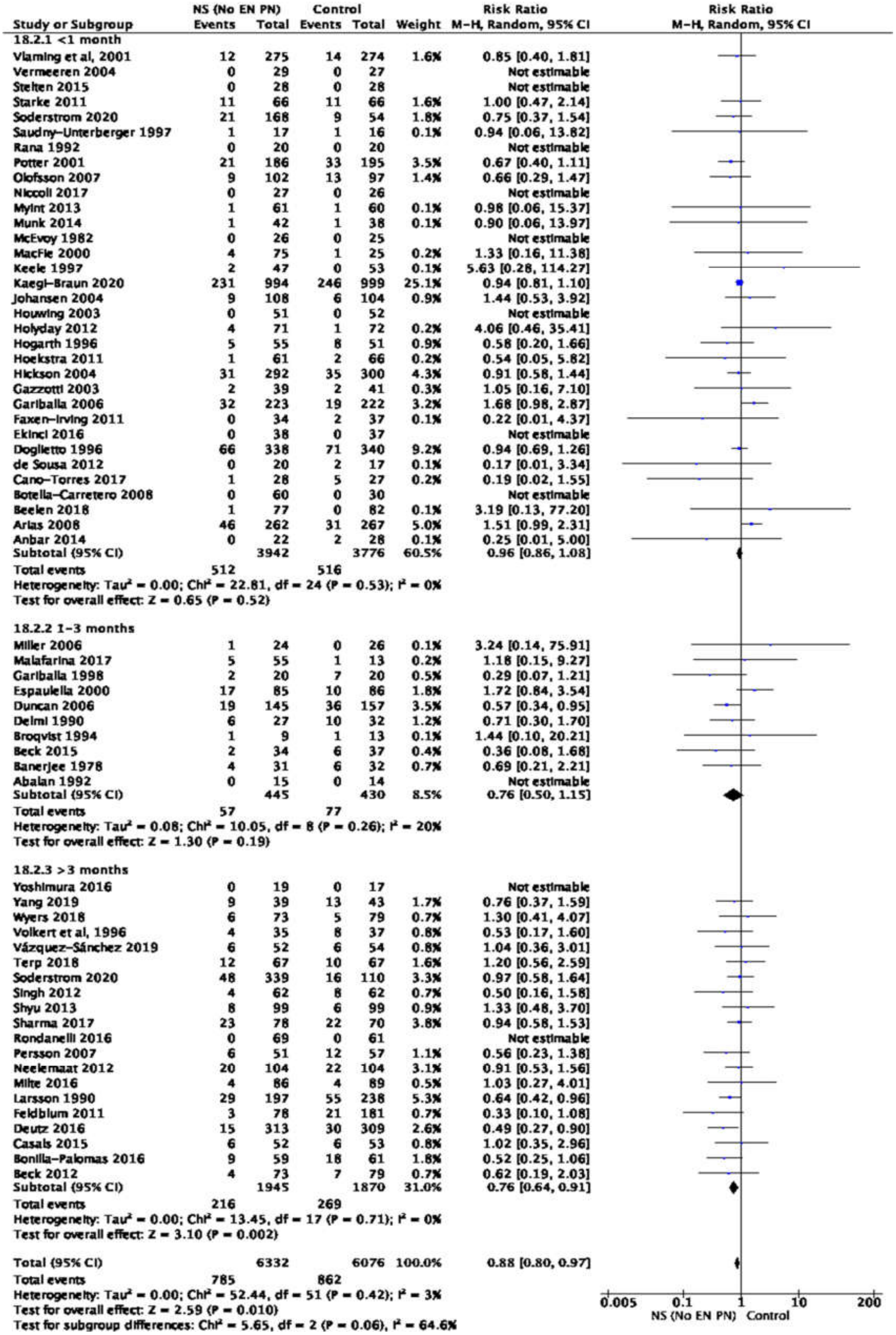

Figure S1. Subgroup analyses of interventions based on the length of the intervention for 6-month mortality.

Supplementary Table S2. Studies Included in Meta-Analysis

Study	Year	Study Type (RCT =1, NRSI = 2, cRCT = 3)	Control	Intervention	Complexity of Intervention (Simple = 1, Complex = 2)
Abalan F, Manciet G, Dartigues JF, Decamps A, Zapata E, Saumtally B, et al. Nutrition and SDAT. Biological Psychiatry 1992;31(1):103-5.	1992	1	1. Standard hospital diet	1. Standard hospital diet<br>2. Oral nutrients (multivitamin, magnesium, calcium, phosphorus, L-tryptophan, Trace elements, B1/6/12, Folic Acid)<br>3. ONS x 2 serves/d (400kcal/d and Protein 22.5g/d)	1
Anbar R, Beloosesky Y, Cohen J, Madar Z, Weiss A, Theilla M,et al. Tight calorie control in geriatric patients following hip fracture decreases complications: a randomized, controlled study. Clinical Nutrition 2014;33(1):23-8.	2014	1	1. Standard care	1. Provision of energy with a goal determined by repeated REE measurements using indirect calorimetry<br>2. Additional ONS if energy not met (~220kcal/d extra)	2
Arias S, Bruzzone I, Blanco V, Inchausti M, Garcia F, Casavieja G, et al. Identification and early nutritional support in hospitalized malnourished patients. Nutricion Hospitalaria 2008;23(4):348-53.	2008	1	1. Standard hospital diet	1. Standard hospital diet and ONS	1
Banerjee AK, Brocklehurst JC, Wainwright H, Swindell R. Nutritional status of long-stay geriatric in-patients: effects of a food supplement (Complan). Age and Ageing 1978;7(4):237-43.	1978	1	1. Dietary education	1. ONS (265kcal/18.6g protein)<br>2. Dietitian spent time (5 days) in the ward looking after patients	1
Barton AD, Beigg CL, Macdonald IA, Allison SP. A recipe for improving food intakes in elderly hospitalized patients. Clinical Nutrition 2000;19(6):451-4.	2000	2	NA	1. Energy-enriched menu (Addition of 200 kcal/d and protein 5 g/d)<br>2. Addition of a cooked breakfast (200 kcal/d and protein 12 g/d per day)	1
Beck A, Andersen UT, Leedo E, Jensen LL, Martins K, Quvang M, et al. Does adding a dietician to the liaison team after discharge of geriatric patients improve nutritional outcome: a randomised controlled trial. Clin Rehabil 2015 Nov;29(11):1117-28.	2015	1	1. Discharge Liaison-Team visit without dietitian	1. Pre-discharge, dietitians evaluated the need for oral supplements and prescribed them as necessary.<br>2. Post-discharge, individualized assessments were conducted, focusing on diet, activity, and weight to inform a personalized nutrition care plan, which could involve coordinating with meal providers for high-energy, protein-rich meals.<br>3. A total of three home visits were scheduled to explore options for supplements and micronutrients, such as vitamin D and	2

Study	Year	Study Type (RCT =1, NRSI = 2, cRCT = 3)	Control	Intervention	Complexity of Intervention (Simple = 1, Complex = 2)
				calcium, partially covered by health insurance, to optimize nutritional intake.	
Beck AM, Kjær S, Hansen BS, Storm RL, Thal-Jantzen K, Bitz C.Follow-up home visits with registered dietitians have a positive effect on the functional and nutritional status of geriatricmedical patients after discharge: a randomized controlled trial.Clinical Rehabilitation 2012; 27(6):483–93.	2012	1	1. Three follow-up visits by general practitioners alone.	1. Patients received three home visits for individualized nutritional counselling by a registered dietitian, complemented by three follow-up visits from general practitioners.<br>2. Strategies to optimize nutritional status included supplementation with energy- and protein-dense meals, ONS, and micronutrient recommendations such as vitamin D and calcium.	2
Beelen J, Vasse E, Janssen N, Janse A, de Roos NM, de Groot LCPGM. Protein-enriched familiar foods and drinks improve protein intake of hospitalized older patients: A randomized controlled trial. Clin Nutr. 2018 Aug;37(4):1186-1192	2018	1	1. Standard energy and protein rich hospital menu	1. Received various protein-enriched products replacing regular products or added to the menu	2
Bell JJ, Bauer JD, Capra S, Pulle RC. Multidisciplinary, multimodal nutritional care in acute hip fracture inpatients – results of a pragmatic intervention. Clin Nutr. 2014;33(6):1101-1107.	2014	2	NA	1. Nutrition assistants implemented a twice-weekly review protocol with scripted high-calorie supplements and selective mid-meals.<br>2. Champions led a multimedia strategy, including posters and electronic media, to boost nutritional awareness.<br>3. The strategy also featured in-services and documentation to educate patients, carers, and the multidisciplinary team.	2

Study	Year	Study Type (RCT =1, NRSI = 2, cRCT = 3)	Control	Intervention	Complexity of Intervention (Simple = 1, Complex = 2)
Bonilla-Palomas JL, et al. Nutritional intervention in malnourished hospitalized patients with heart failure. Arch Med Res 2016;47(7):535-40.	2016	1	1. Standard care	1. A 6-month nutritional intervention was led by a physician specialist and a nutritionist, starting at admission.<br>2. The protocol focused on diet optimization, specific recommendations, and supplement prescriptions when goals were unmet.<br>3. Supplement choices were tailored to patient requirements and co-morbidities.	2
Botella-Carretero JI, Iglesias B, Balsa JA, Zamarron I, Arrieta F, Vazquez C. Effects of oral nutritional supplements in normally nourished or mildly undernourished geriatric patients after surgery for hip fracture: a randomized clinical trial. Journal of Parenteral and Enteral Nutrition 2008;32(2):120-8.	2008	1	1. Standard diet	1. Intervention 1: Standard diet + Extra Protein 36g/d<br>2. Intervention 2: Standard diet + Extra 500kcal/d + + Extra Protein 36g/d	1
Broqvist M, et al. Nutritional assessment and muscle energy metabolism in severe chronic congestive heart failure–effects of long-term dietary supplementation. Eur Heart J 1994;15(12):1641-50.	1994	1	1. Placebo ONS	1. ONS (500 ml/d, Protein 30 g/d, 750 kcal/d)	1
Brown, Helen; Jones, Lucy (2009). The role of dining companions in supporting nursing care. Nursing Standard, 23(41), 40–46. doi:10.7748/ns2009.06.23.41.40.c7049	2009	2	NA	1. Six nursing student volunteers were locally recruited and trained via an interactive workshop led by a speech therapist and dietitian.<br>2. Speech therapists liaised with nurses for initial two weeks to offer volunteer support and guidance.<br>3. Ward nurses handled nutritional care planning and delegated patient feeding.	2
Buys DR, Flood KL, Real K, Chang M, Locher JL. Mealtime assistance for hospitalized older adults - a report on the spoons volunteer program. J Gerontol Nurs 2013;39(9):18- 22.	2013	2	NA	1. Volunteers were trained in three sessions by a unit coordinator and occupational therapist (OT).<br>2. On average, volunteers spent 47.8 minutes per encounter, resulting in cost savings.<br>3. Savings averaged $11.94 per encounter compared to a patient care technician (PCT) and $26.00 compared to a registered nurse (RN).	2

Study	Year	Study Type (RCT =1, NRSI = 2, cRCT = 3)	Control	Intervention	Complexity of Intervention (Simple = 1, Complex = 2)
Campbell KL, Webb L, Vivanti A et al. (2013) Comparison of three interventions in the treatment of malnutrition in hospitalised older adults: a clinical trial. Nutr Diet 70, 325–331.	2013	2	NA	1. Diet Assistants (DAs) at $AU28.10/hr facilitated traditional and mid-meal services, offering up to four energy-enriched snacks per day.<br>2. Snacks provided 70-120 kcal and 0-6 g protein, sourced from a mid-meal trolley of high-protein and/or high-energy options.<br>3. Selections included various items like flavoured milk, chocolate biscuits, and nuts, delivered between main meals.	1
Cano-Torres EA, et al. Impact of nutritional intervention on length of hospital stay and mortality among hospitalized patients with malnutrition: a clinical randomized controlled trial. J Am Coll Nutr 2017;36(4):235-9	2017	1	1. Standard nutritional management according to the Hospital Nutrition Department.	1. Individualized nutrition plan according to energy and protein (1.0–1.5 g/kg) intake requirements<br>2. Dietary advice based on face-to-face interviews with patients and their caregivers or family members.	2
Casals C, García-Agua-Soler N, Vázquez-Sánchez M, Requena-Toro MV, Padilla-Romero L, Casals-Sánchez JL. Randomized Clinical Trial of Nutritional Counselling for Malnourished Hospital Patients. Rev Clin Esp (Barc). 2015;215(6):308-14.	2015	1	1. Standard care	1. Dietary counselling x 3 by case manager nurses during the hospital stay and upon discharge (repeated at first month and subsequently every two months) (n=52)	2
Chen CC-H, Yang Y-T, Lai I-R, Lin B-R, Yang C-Y, Huang J, et al. Three nurse administered protocols reduce nutritional decline and frailty in older gastrointestinal surgery patients: a cluster randomized trial. J Am Med Dir Assoc 2019;20(5):524-9.e3.	2019	3	1. Standard hospital care provided by physicians and nurses on the general surgery wards.	1. Hospital-based mHELP comprising 3 nursing protocols: early mobilization, oral and nutritional assistance, and orienting communication.<br>2. No ONS	2
de Sousa OL, Amaral TF. Three-week nutritional supplementation effect on long-term nutritional status of patients with mild Alzheimer disease. Alzheimer Disease and Associated Disorders 2012;26(2):119-23. [PMID: 21878804]	2012	1	1. Standard dietetic advice	1. ONS x 1 serve/d<br>2. Standard dietetic advice	1
Delmi M, Rapin CH, Bengoa JM, Delmas PD, Vasey H, Bonjour JP. Dietary supplementation in elderly patients with fractured neck of the femur. Lancet 1990;335(8696):1013-6.	1990	1	1. Standard diet	1. ONS x 1 serve/d<br>2. Standard hospital diet	1

Study	Year	Study Type (RCT =1, NRSI = 2, cRCT = 3)	Control	Intervention	Complexity of Intervention (Simple = 1, Complex = 2)
Dennis M, Lewis S, Cranswick G, Forbes J. FOOD: a multicentre randomised trial evaluating feeding policies in patients admitted to hospital with a recent stroke. Health Technology Assessment 2006;10:iii-iv, ix-x, 1-120. Dennis MS, Lewis SC, Warlow C. Routine oral nutritional supplementation for stroke patients in hospital (FOOD): a multicentre randomised controlled trial. Lancet 2005; Vol. 365, issue 9461:755-63.	2005	1	1. Standard diet	1. Three doses of 120 ml 1.5 kcal/ml ONS given daily and prescribed on the drug chart and normal hospital diet until hospital discharge. ONS decanted into a cup for patient<br>2. A wide variety of ONS of different consistencies was sourced for patients	1
Deutz NE, et al. Readmission and mortality in malnourished, older, hospitalized adults treated with a specialized ONS: a randomized clinical trial. Clin Nutr 2016;35(1):18-26.	2016	1	1. Standard-of-care plus placebo supplement	1. Standard-of-care plus ONS with high protein and HMB	1
Dube, L., Paquet, C., Ma, Z., Kergoat, M., Ferland, G., 2007. Nutritional implications of patient-provider interactions in hospital settings: evidence from a within subject assessment of mealtime exchanges and food intake in elderly patients. Eur. J. Clin. Nutr. 61, 664–672.	2007	2	NA	1. Communal Dining Room<br>2. All participants were assigned to one of six tables, where they ate all their meals in the company of up to three fellow patients<br>3. A team of care providers (nurses and orderlies) was present to perform meal service and provide meal assistance	1
Duncan, D.G., Beck, S.J., Hood, K., Johansen, A., 2006. Using DAs to improve the outcome of hip fracture: a randomised controlled trial of nutritional support in an acute trauma ward. Age Ageing 35, 148–153.	2006	1	1. Conventional pattern of nurse- and dietitian-led care	1. Dietetic assistant	1

Study	Year	Study Type (RCT =1, NRSI = 2, cRCT = 3)	Control	Intervention	Complexity of Intervention (Simple = 1, Complex = 2)
Ekinci, O., Yanik, S., Terzioğlu Bebitoğlu, B., Yilmaz Akyüz, E., Dokuyucu, A., & Erdem, Ş. (2016). Effect of Calcium β-Hydroxy-β-Methylbutyrate (CaHMB), vitamin D, and protein supplementation on postoperative immobilization in malnourished older adult patients with hip fracture: A randomized controlled study. Nutrition in Clinical Practice, 31, 829–835. https ://doi.org/10.1177/0884533616629628	2016	1	1. Standard postoperative nutrition	1. ONS (high protein + HMB) x 2 serves/d in addition to standard postoperative nutrition	1
Espaulella J, Guyer H, Diaz-Escriu F, Mellado-Navas JA, Castells M, Pladevall M. Nutritional supplementation of elderly hip fracture patients. A randomized, double-blind, placebo controlled trial. Age and Ageing 2000;29(5):425-31.	2000	1	1. Placebo ONS (152kcal 0g protein) x 1 serve/d	1. ONS (149kcal/d 20g/d protein Ca Vit D3) x 1 serve/d	1
Faxen-Irving G, Cederholm T. Energy dense oleic acid rich formula to newly admitted geriatric patients--feasibility and effects on energy intake. Clinical Nutrition 2011; 30(2):202-8.	2011	1	1. Standard care	1. 30 ml fat emulsion (Calogen) x 3 serves/d (420 kcal/d)	1
Feldblum I, et al. Individualized nutritional intervention during and after hospitalization: the nutrition intervention study clinical trial. J Am Geriatr Soc 2011;59(1):10-7.	2011	1	1. One group had a single hospital meeting with a dietitian and received advised supplementation during hospitalization.<br>2. A second group received standard care with unreported supplement intake.<br>3. Both groups were later combined to form a single control group for the study.	1. Patients received one inpatient and two home dietary counseling sessions by a Dietitian, with follow-up assessments at 3 and 6 months post-discharge by a Coordinator.<br>2. Coordinator also made six phone calls for monitoring, and patients were prescribed 1-2 servings of 1.5 kcal/ml supplements up to 6 months, including options like liquid or pudding.<br>3. When micronutrient intake was below 75% of the Daily Recommended Intake (DRI), appropriate vitamin and mineral supplements were provided, with the amount being unspecified.	2

Study	Year	Study Type (RCT =1, NRSI = 2, cRCT = 3)	Control	Intervention	Complexity of Intervention (Simple = 1, Complex = 2)
Gariballa S, et al. A randomized, double-blind, placebo-controlled trial of nutritional supplementation during acute illness. Am J Med 2006;119(8): 693-9.	2006	1	1. Normal hospital diet plus a placebo daily for 6 weeks.	1. Normal hospital diet plus ONS 400ml/d for 6 weeks	1
Gariballa SE, Parker SG, Taub N, Castleden CM. A randomized,controlled, a single-blind trial of nutritional supplementation after acute stroke. Journal of Parenteral and Enteral Nutrition 1998;22(5):315-9.	1998	1	1. Standard care	1. Hospital diet plus a twice daily ONS of >400 mL (600 kcal/d and protein 20 g/d) at 3:00 PM and 8:00 PM each day for 4 weeks or until death or discharge.	1
Gazzotti C, et al. Prevention of malnutrition in older people during and after hospitalisation: results from a randomised controlled clinical trial. Age Ageing 2003;32(3):321-5.	2003	1	1. Standard care	1. 200 ml sweet or salty ONS twice daily (500 kcal/d and protein 21g/d) throughout hospitalisation and convalescence.	1
Hayward J. Ward nutrition coordinators to improve patient nutrition in hospital. Br J Nurs. 2003;12(18):1081-1089.	2003	2	NA	1. Each ward had a full-time nutrition coordinator who underwent a one-week multidisciplinary induction program, including training with dietitians, caterers, speech and occupational therapists, and a nutrition nurse specialist.<br>2. The program encompassed various aspects such as therapeutic diets, feeding aids, patient assessment, and documentation, featuring hands-on activities like tasting sessions and swallowing assessments.<br>3. Ongoing support for the coordinators was agreed to be provided by both the clinical team and the nutrition nurse specialist.	2
Hickson M, Bulpitt C, Nunes M, Peters R, Cooke J, Nicholl C, et al. Does additional feeding support provided by health care assistants improve nutritional status and outcome in acutely ill older in-patients? a randomised control trial. Clin Nutr 2004;23(1):69-77	2004	1	1. Standard care	1. Patients received additional nutritional care from a trained Health Care Assistant (HCA) upon randomization.<br>2. All patients were prescribed medical and nutritional therapy as standard care.<br>3. The only variable was the level of feeding support provided by the trained HCA.	1

Study	Year	Study Type (RCT =1, NRSI = 2, cRCT = 3)	Control	Intervention	Complexity of Intervention (Simple = 1, Complex = 2)
Hickson, M., Connolly, A., Whelan, K., 2011. Impact of protected mealtimes on ward mealtime environment, patient experience and nutrient intake in hospitalised patients. J. Hum. Nutr. Diet. 24, 370–374. doi:http://dx.doi.org/10.1111/j.1365-277X.2011.01167.x.	2011	2	NA	1. Protected Mealtimes were implemented via a guideline document and disseminated through the Nursing directorate.<br>2. Notices were posted on the intranet to inform staff and stakeholders.<br>3. Large signs were displayed to indicate when Protected Mealtimes were in progress.	2
Hoekstra JC, Goosen JH, de Wolf GS, Verheyen CC. Effectiveness of multidisciplinary nutritional care on nutritional intake, nutritional status and quality of life in patients with hip fractures: a controlled prospective cohort study. Clin Nutr 2011 Aug;30(4):455-61.	2011	1	1. Standard nutritional care	1. Multidisciplinary nutritional care that focused on nutritional support during hospitalisation and a transfer of nutritional care after discharge.	2
Hogarth MB, et al. Nutritional supplementation in elderly medical in-patients: a double-blind placebo-controlled trial. Age Ageing 1996;25(6):453-7.	1996	1	1. Placebo energy and active vitamin supplementation<br>2. Placebo energy and placebo vitamin supplementation.	1. Active energy and active vitamin supplementation<br>**2. Active energy and placebo vitamin supplementation**	1
Holst M, Beermann T, Mortensen MN, Skadhauge LB, Køhler M, Lindorff-Larsen K, et al. Optimizing protein and energy intake in hospitals by improving individualized meal serving, hosting and the eating environment. Nutrition 2017;34:14-20.	2017	2	NA	1. Health Care Professionals (HCP) including nursing staff and kitchen hostesses received training as part of the intervention.<br>2. The training, conducted by the nutrition support team and hospital nutrition board, covered topics such as aesthetic meal serving, portion sizes, and patient assistance during meals.<br>3. Special emphasis was placed on catering to individual preferences and offering targeted meal assistance.	2

Study	Year	Study Type (RCT =1, NRSI = 2, cRCT = 3)	Control	Intervention	Complexity of Intervention (Simple = 1, Complex = 2)
Holyday M, et al. Malnutrition screening and early nutrition intervention in hospitalised patients in acute aged care: a randomised controlled trial. J Nutr Health Aging 2012;16(6):562-8.	2012	1	1. Usual nutrition care provided.<br>2. Control group only seen by the clinical dietitian if and when referred by medical or other health professionals, and if referred, the same care plan was implemented as for the intervention patients.	1. The Malnutrition Care Plan (MCP) included texture-modified and fortified hospital meals, prescription of nutrient-dense supplements, and meal assistance flagging by ward staff.<br>2. Education on optimizing nutrition was provided to patients and carers, and referrals were made for discharge planning.<br>3. The MCP was customized according to individual patient needs, based on the clinical dietitian's assessment and prescription.	2
Houwing RH, Rozendaal M, Wouters-Wesseling W, Beulens JW, Buskens E, Haalboom JR. A randomised, double-blind assessment of the effect of nutritional supplementation on the prevention of pressure ulcers in hip-fracture patients. Clinical Nutrition 2003;22(4):401-5.	2003	1	1. Placebo ONS (non-caloric, water-based drink containing only sweeteners, colourants and flavourings)	1. 400ml high-protein ONS enriched with arginine, zinc and antioxidants starting immediately postoperatively for a period of 4 weeks or until discharge.	1
Howson FFA, Robinson SM, Lin SX, Orlando R, Cooper C, Sayer AAP, et al. Can trained volunteers improve the mealtime care of older hospital patients? An implementation study in one English hospital. BMJ Open 2018;8(8).	2018	2	NA	1. After competency assessment, volunteers were met by the research team during their next two mealtime shifts to address any concerns and ensure acclimation to the ward.<br>2. Semi-annual 'coffee and cake' meetings were held to discuss implementation progress and address volunteer concerns; volunteers typically assisted 2-3 patients per session, including feeding one.<br>3. Implementation costs over the 15-month study included 13 training sessions, individual competency sessions, and one hour of administrative support per volunteer for coordination.	2
Huang CS, Dutkowski K, Fuller A, Walton K. Evaluation of a pilot volunteer feeding assistance program: influences on the dietary intakes of elderly hospitalised patients and lessons learnt. J Nutr Health Aging 2015;19(2): 206-10.	2015	2	NA	1. Volunteers trained by dietetics, speech pathology and nursing staff	2

Study	Year	Study Type (RCT =1, NRSI = 2, cRCT = 3)	Control	Intervention	Complexity of Intervention (Simple = 1, Complex = 2)
Huxtable, S., Palmer, M., 2013. The efficacy of protected mealtimes in reducing mealtime interruptions and improving mealtime assistance in adult inpatients in an Australian Hospital. Eur. J. Clin. Nutr. 67, 904–910.	2013	2	NA	1. Staff training occurred approximately 5-7 months before and 5-6 months after the intervention, with volunteers aiding mealtime on 4 wards post-implementation. The nursing unit manager acted as an informal champion.<br>2. Four in-service educational sessions were conducted between 1-13 months post-Protected Mealtimes Program (PMP) implementation, focusing on effective meal tray delivery and patient assistance.<br>3. Speech Pathologists restructured clinics to allocate mealtime appointments for specific patient groups, and volunteers were deployed up to 8 months post-PMP to assist with meals.	2
Huynh DT, et al. Effects of oral nutritional supplementation in the management of malnutrition in hospital and post-hospital discharged patients in India: a randomised, open-label, controlled trial. J Hum Nutr Diet 2015;28(4): 331-43.	2015	1	1. Dietary education	1. Prescription of nutritional supplements and dietary education were administered; 100% of participants received the supplements.<br>2. Dietary counselling was provided by a Dietitian.<br>3. Three sessions of dietary counselling were conducted at baseline, week 4, and week 8 of the intervention.	2
Johansen N, Kondrup J, Plum LM, Bak L, Norregaard P, Bunch E, et al. Effect of nutritional support on clinical outcome in patients at nutritional risk. Clinical Nutrition 2004;23(4):539-50.	2004	1	1. Received nutritional care according to the usual procedure of the department.	1. The team provided daily attention to motivate both patients and staff for nutritional adherence.<br>2. Tailoring of the nutritional plan involved estimating protein and energy requirements, and coordinating food orders with patients.<br>3. The team also provided guidance on transitioning to alternative feeding methods such as tube feeding or parenteral nutrition when necessary.	2
Keele AM, Bray MJ, Emery PW, Duncan HD, Silk DBA. Two phase randomised controlled clinical trial of postoperative oral dietary supplements in surgical patients. Gut 1997;40(3):393-9.	1997	1	1. Standard hospital diet only	1. Standard hospital diet supplemented ad libitum with ONS (Fortisip, Nutricia, Holland) both in and outpatient	1

Study	Year	Study Type (RCT =1, NRSI = 2, cRCT = 3)	Control	Intervention	Complexity of Intervention (Simple = 1, Complex = 2)
Larsson J, Unosson M, Ek AC, Nilsson L, Thorslund S, Bjurulf P. Effect of dietary supplement on nutritional status and clinical outcome in 501 geriatric patients--a randomised study. Clin Nutr. 1990 Aug;9(4):179-84. doi: 10.1016/0261-5614(90)90017-m. PMID: 16837353.	1990	1	1. Standard hospital diet	1. ONS (400 kcal/d) as well as a standard hospital diet	1
Lassen KO, Grinderslev E, Nyholm R. Effect of changed organisation of nutritional care of Danish medical inpatients. BMC Health Serv Res. 2008;8(1):1-13.	2008	2	NA	1. A specialist role for Nutritional Health Care Assistants was established to ensure the nutritional and fluid needs of at-risk patients are met post-assessment.<br>2. Training involved a one-month program aimed at upskilling Social and Healthcare Assistants to the level of Nutritional and Healthcare Assistants.<br>3. The focus of the role and training was on providing targeted nutrition care to patients identified as nutritionally at-risk	2
Lassen KO, Kruse F, Bjerrum M, Jensen L, Hermansen K. Nutritional care of Danish medical inpatients: effect on dietary intake and the occupational groups' perspectives of intervention. Nutr J 2004;3(1):12.	2004	2	NA	1. The kitchen introduced new diet regimens during the study period as an additional variable.<br>2. Nurses received four training meetings to acquaint them with new forms and usage protocols; the timing of these meetings was not specified.<br>3. Weekly support was provided by investigators to the nursing staff, though the frequency and duration were not detailed.	2
MacFie J, Woodcock NP, Palmer MD, Walker A, Townsend S, Mitchell CJ. Oral dietary supplements in pre- and postoperative surgical patients: a prospective and randomized clinical trial. Nutrition 2000;16(9):723-8.	2000	1	1. Group IV did not receive any supplements.	1. Group I received oral dietary supplements (ODS) both pre- and postoperatively in addition to their normal diet.<br>2. Group II was administered ODS exclusively in the preoperative period.<br>3. Group III received ODS only during the postoperative period.	2

Study	Year	Study Type (RCT =1, NRSI = 2, cRCT = 3)	Control	Intervention	Complexity of Intervention (Simple = 1, Complex = 2)
Malafarina V, Uriz-Otano F, Malafarina C, Martinez JA, Zulet MA. Effectiveness of nutrition supplementation on sarcopenia and recovery in hip fracture patients. A multi-centre randomized trial. Maturitas. 2017 Jul;101:42-50. doi: 10.1016/j.maturitas.2017.	2017	1	1. Standard diet + rehabilitative therapy	1. Standard diet plus ONS x 2 serves/d (with HMB) + rehabilitative therapy	2
Manning F, Harris K, Duncan R, Walton K, Bracks J, Larby L, et al. Additional feeding assistance improves the energy and protein intakes of hospitalised elderly patients. A health services evaluation. Appetite 2012;59(2):471-7.	2012	2	NA	1. Nurses spent a mean time of 6 min with patients during mealtimes providing assistance and only 4.7 min at lunchtime. Volunteers provided more than double this amount of time	1
Maunder K, Lazarus C, Walton K, Williams P, Ferguson M, Beck E. Energy and protein intake increases with an electronic bedside spoken meal ordering system compared to a paper menu in hospital patients. Clin Nutr ESPEN. 2015;10(4):e134-e139.	2015	2	1. Paper Menu	1. Electronic bedside spoken meal ordering systems (BMOS)	1
McEvoy AW, James OF. The effect of a dietary supplement (build-up) on nutritional status in hospitalized elderly patients. Hum Nutr Appl Nutr 1982;36(5):374-6.	1982	1	1. Standard care	1. ONS	1
Miller MD, Crotty M, Whitehead C, Bannerman E, Daniels LA. Nutritional supplementation and resistance training in nutritionally at risk older adults following lower limb fracture: a randomized controlled trial. Clin Rehabil 2006 Apr;20(4):311-23.	2006	1	1. Attention control plus usual care and general nutrition and exercise advice	1. Group I: ONS (6.3 kJ/mL) for six weeks<br>2. Group II: tri-weekly resistance training for 12 weeks<br>3. Group III: combined treatment for 24 weeks	2

Study	Year	Study Type (RCT =1, NRSI = 2, cRCT = 3)	Control	Intervention	Complexity of Intervention (Simple = 1, Complex = 2)
Milte R, Miller MD, Crotty M, Mackintosh S, Thomas S, Cameron ID, et al. Cost-Effectiveness of Individualized Nutrition and Exercise Therapy for Rehabilitation Following Hip Fracture. J Rehabil Med. 2016;48(4):378-85.	2016	1	1. Attention control for 6 months.	1. A six-month individualized nutrition and exercise program was implemented for each participant.<br>2. The dietary component involved tailored counselling on meal timing, size, and frequency, as well as recommendations for nutrient-rich foods and recipes.<br>3. Referrals to community meal programs and provision of commercial oral nutritional supplements or protein powders were made as deemed appropriate.	2
Munk T, et al. Positive effect of protein-supplemented hospital food on protein intake in patients at nutritional risk: a randomised controlled trial. J Hum Nutr Diet 2014;27(2): 122-32.	2014	1	1. Standard hospital food service	1. Participants were provided with a targeted food concept featuring an a la carte menu of small, energy-dense dishes, supplemented with high-quality protein powder.<br>2. The dishes could be ordered via telephone by patients, ward staff, or research assistants.<br>3. The ordered dishes were presented and served by kitchen staff utilizing a 'room service' approach.	1
Munk T, Seidelin W, Rosenbom E et al. (2013) A 24-h a la carte food service as support for patients at nutritional risk: a pilot study. J Hum Nutr Diet 26, 268–275.	2013	2	NA	1. A 'room service' approach was employed where dishes could be ordered by patients, ward staff, or research assistants from 07:00 to 20:00, seven days a week, with delivery guaranteed within 20 minutes of placing an order.<br>2. To ensure uninterrupted nutritional intake, especially during weekends with reduced ward staff, patients had the option to place orders 48 hours in advance.<br>3. The offered dishes were energy and protein-enriched, fortified with a high-quality milk protein powder, and served in addition to the standard diet. These dishes contained 6.1-11.5 g of protein and 2.5-19.8 kJ/g of energy.	2
Myint, M. W. W., Wu, J., Wong, E., Chan, S. P., To, T. S. J., Chau, M. W.R., Au, K. S. D. (2013). Clinical benefits of oral nutritional supplementation for elderly hip fracture patients: A single blind randomised controlled trial. Age and Ageing, 42, 39–45. https ://doi.org/10.1093/ageing/afs078	2013	1	1. Standard hospital diet	1. ONS + diet (n=65) for a maximum duration of 4 weeks	1

Study	Year	Study Type (RCT =1, NRSI = 2, cRCT = 3)	Control	Intervention	Complexity of Intervention (Simple = 1, Complex = 2)
Neelemaat F, Bosmans JE, Thijs A, Seidell JC, van Bokhorstde van der Schueren MA. Post-discharge nutritional support in malnourished elderly individuals improves functional limitations. Journal of the American Medical Directors Association 2011;12(4):295-301	2011	1	1. Standard Care Treating physician may prescribe supplements.	1. Participants received standardized nutritional support from hospital admission until 3 months post-discharge, beginning with an energy- and protein-enriched diet during their in-hospital stay.<br>2. Nutritional supplementation included two servings of supplements daily, with 100% of patients initially on supplements and 84% continuing at the 3-month mark, alongside 400 IU vitamin D3 and 500 mg calcium per day.<br>3. After discharge, patients received bi-weekly phone counselling by a dietitian for a total of six sessions.	2
Niccoli S, Kolobov A, Bon T, Rafilovich S, Munro H, Tanner K, Pearson T, Lees SJ. Whey Protein supplementation Improves Rehabilitation Outcomes in Hospitalized Geriatric Patients: A Double Blinded, Randomized Controlled Trial. J Nutr Gerontol Geriatr. 2017 Oct-Dec;36(4):149-165.	2017	1	1. Received hot cereal and milk products without the whey protein.	1. Participants were given an oral dietary product containing a total of 24 g of whey protein per day, in addition to their usual diet.<br>2. The whey protein supplement (WPS) was incorporated into the participants' daily meals: 9 g mixed into hot cereal at breakfast and 7.5 g per drink at lunch and dinner.	1
Olofsson, B, Stenvall, M, Lundstrom, M, Svensson, O, Gustafson, Y., 2007. Malnutrition in hip fracture patients: an intervention study. J. Clin. Nurs. 16 (11), 2027–2038. doi: 10.1111/j.1365-2702.2006.01864.x .	2007	1	1. Standard hospital diet	1. A nutritional journal was used to identify nutritional deficiencies, and protein-enriched meals were provided for a minimum of four days postoperatively.<br>2. At least two nutritional and protein drinks were administered daily throughout the hospitalization period.<br>3. Additional factors affecting the patient's nutritional status were also identified and managed.	2
Pedersen PU, Tewes M, Bjerrum M. Implementing nutritional guidelines the effect of systematic training for nurse nutrition practitioners. Scand J Caring Sci 2012; 26(1): 178-85.	2012	2	NA	1. Nurses underwent comprehensive training featuring five modules, each lasting 3–4 days.<br>2. Training modules included a blend of nutritional education and patient involvement in nutritional care, along with implementation strategies and change theories.<br>3. Each training day consisted of six hours of instruction.	2

Study	Year	Study Type (RCT =1, NRSI = 2, cRCT = 3)	Control	Intervention	Complexity of Intervention (Simple = 1, Complex = 2)
Persson M, Hytter-Landahl A, Brismar K, Cederholm T. Nutritional Supplementation and Dietary Advice in Geriatric Patients at Risk of Malnutrition. Clin Nutr. 2007;26(2):216-24.	2007	1	1. Given brief written dietary advice. (n=25)	1. Participants received two dietary counselling sessions by a dietitian: one before hospital discharge and another one week after.<br>2. All participants were prescribed 1-2 servings of nutritional supplements along with a daily multivitamin.<br>3. Dietitian maintained phone contact at three specific time-points: 1–2 weeks post-discharge, mid-study, and one week prior to follow-up.	2
Porter J, Haines T, Truby H. The efficacy of Protected Mealtimes in hospitalised patients: a stepped wedge cluster randomised controlled trial. BMC Med. 2017;15(1):25. doi:10.1186/s12916-017-0780-1	2017	3	1. Standard care	1. The translation project employed four primary intervention approaches: education, restrictions, environmental restructuring, and enablement.<br>2. A variety of strategies were collaboratively developed with relevant staff to facilitate optimal implementation of Protected Mealtimes.<br>3. The aim was to create a multi-faceted approach that could address various challenges and ensure successful implementation.	2
Potter JM, Roberts MA, McColl JH, Reilly JJ. Protein energy supplements in unwell elderly patients - a randomized controlled trial. JPEN Journal of Parenteral and Enteral Nutrition 2001;25(6):323-9.	2000	1	1. Standard care	1. 1.5 kcal/mL supplement aimed to provide 22.5g protein and 540 kcal energy per day.<br>2. Administered thrice daily in 120 mL doses. Initiated within 48 hours of admission. 2. Nurses observed and recorded compliance during supplement administration, as with other medications.<br>3. Supplementation continued until discharge, death, or transfer to long-term care.	1
Poulsen I, Vendel Petersen H, Rahm Hallberg I, Schroll M. Lack of nutritional and functional effects of nutritional supervision by nurses: a quasi-experimental study in geriatric patients. Scand J Food Nutr. 2007;51(1):6-12. doi:10.1080/17482970701256245	2007	2	NA	1. Nutrition assessment incorporated into nursing plans.<br>2. Three-day dietary tracking by nurses.<br>3. Nurses trained via 90-min lectures.	2

Study	Year	Study Type (RCT =1, NRSI = 2, cRCT = 3)	Control	Intervention	Complexity of Intervention (Simple = 1, Complex = 2)
Rana SK, Bray J, Menzies-Gow N, Jameson J, James JJP, Frost P, et al. Short term benefits of post-operative oral dietary supplements in surgical patients. Clinical Nutrition 1992;11(6):337-44. [PUBMED: 16840018]	1992	1	1. Standard diet	1. Standard hospital diet supplemented ad libitum with a ONS (1.5kcal/ml)	1
Roberts HC, De Wet S, Porter K, Rood G, Diaper N, Robison J, et al. The feasibility and acceptability of training volunteer mealtime assistants to help older acute hospital inpatients: the Southampton Mealtime Assistance Study. J Clin Nurs 2014;23(21-22):3240-9.	2014	2	NA	1. Volunteers were trained in half-day sessions, designed by a speech and language therapist (SALT) and a dietitian.<br>2. Training was conducted in groups of up to eight volunteers, spanning seven different half-day sessions.<br>3. Post-training, each volunteer's competency was assessed by the speech and language therapist during a mealtime, approximately lasting 30 minutes.	2
Roberts HC, Pilgrim AL, Jameson KA, Cooper C, Sayer AA, Robinson S. The impact of trained volunteer mealtime assistants on the dietary intake of older female in-patients: the Southampton Mealtime Assistance Study. J Nutr Health Aging 2017;21(3):320-8.	2017	2	NA	1. comprehensive training program for volunteers was designed by a multidisciplinary team comprising a speech and language therapist and a dietitian.<br>2. The training was segmented into seven half-day sessions, with each session accommodating groups of up to eight volunteers.<br>3. Following the training, a formal competency assessment was conducted for each volunteer by the speech and language therapist, focusing on their aptitude in fulfilling the roles and responsibilities of a mealtime assistant. The evaluation lasted approximately 30 minutes per volunteer.	2
Robinson S, Clump D, Weitzel T, Henderson L, Lee K, Schwartz C, et al. The memorial meal mates: a program to improve nutrition in hospitalized older adults. Geriatr Nurs 2002;23(6):332-5.	2002	2	NA	1. The volunteers were provided a 3-hr in-service taught by an interdisciplinary team composed of 4 nurses, a dietitian, a ST, and an occupational therapist.	2
Robison J, Pilgrim AL, Rood G, Diaper N, Elia M, Jackson AA, et al. Can trained volunteers make a difference at mealtimes for older people in hospital? A qualitative study of the views and experience of nurses, patients, relatives and volunteers in the Southampton Mealtime Assistance Study. Int J Older People Nurs 2015;10(2):136-45.	2015	2	NA	1. Volunteers trained in half day sessions 7 times developed by Swallowing Therapist and Dietitian	2

Study	Year	Study Type (RCT =1, NRSI = 2, cRCT = 3)	Control	Intervention	Complexity of Intervention (Simple = 1, Complex = 2)
Rondanelli M, Klersy C, Terracol G, Talluri J, Maugeri R, Guido D, Faliva MA, Solerte BS, Fioravanti M, Lukaski H, Perna S. Whey protein, amino acids, and vitamin D supplementation with physical activity increases fat-free mass and strength, functionality, and quality of life and decreases inflammation in sarcopenic elderly. Am J Clin Nutr. 2016 Mar;103(3):830-40.	2016	1	1. Placebo with concurrent with regular, controlled physical activity (n=61)	1. Supplementation with whey protein (22 g), essential amino acids (10.9 g, including 4 g leucine), and vitamin D [2.5 mg (100 IU)] concurrent with regular, controlled physical activity	2
Rufenacht U, et al. Nutritional counselling improves quality of life and nutrient intake in hospitalized undernourished patients. Nutrition 2010;26(1): 53-60.	2010	1	1. ONS only	1. Patients were individually counseled by a dietitian following standardized procedures equivalent to standard care, leading to the creation of personalized nutritional plans.<br>2. The nutritional plans included various interventions such as food enrichment with energy and/or protein, utilizing supplements like maltodextrin, oil, and powdered protein.<br>3. ONS + standard hospital meals	2
Ryan M, et al. Oral nutritional supplements differing in fat and carbohydrate content: effect on the appetite and food intake of undernourished elderly patients. Clin Nutr 2004;23(4):683-9.	2004	2	NA	1. ONS	1
Salisbury LG, Merriweather JL, Walsh TS. The development and feasibility of a ward-based physiotherapy and nutritional rehabilitation package for people experiencing critical illness. Clin Rehabil. 2010;24(6):489-500.	2010	2	NA	1. The enhanced nutritional rehabilitation program featured assistance at mealtimes, meticulous monitoring of supplement delivery and consumption, as well as frequent food charts to track and ensure adequate oral intake.<br>2. Generic rehabilitation assistants conducted one to two daily visits to facilitate the dietary and therapeutic tasks, although the duration of each visit was unspecified.<br>3. To equip the generic rehabilitation assistants with the necessary competencies, discipline-specific training was provided. However, details about the duration and quality of this training were not specified.	2

Study	Year	Study Type (RCT =1, NRSI = 2, cRCT = 3)	Control	Intervention	Complexity of Intervention (Simple = 1, Complex = 2)
Saudny-Unterberger H, Martin JG, Gray-Donald K. Impact of nutritional support on functional status during an acute exacerbation of chronic obstructive pulmonary disease. Am J Respir Crit Care Med 1997;156(3 Pt 1):794-9	1997	1	1. Standard diet (n=16)	1. In the supplemental feeding group, patients received additional oral supplements like Ensure, Ensure Plus, or a variety of puddings along with their regular hospital tray.<br>2. The objective was to assure a caloric intake of at least 1.5 times the Resting Energy Expenditure (REE) for patients with a normal Body Mass Index (BMI) ranging from 20 to 27.<br>3. For those with a BMI below 20, the caloric intake target was set at a higher level of at least 1.7 times the REE.	2
Schuetz P, et al. Individualised nutritional support in medical inpatients at nutritional risk: a randomised clinical trial. Lancet 2019;393 (10188):2312-21. Kaegi-Braun, et al. 2020	2020	1	1. Provided with standard hospital food, no nutritional consultation and no recommendation for additional nutritional support	1. An individualized nutritional plan was crafted by a dietitian for each patient, addressing elements like food preferences, food fortification, inter-meal snacks, and nutritional supplements.<br>2. In the hospital setting, 91% of the patients in the intervention group received oral nutritional supplements as part of their tailored nutritional care.<br>3. Upon discharge, 24% of the patients in the intervention group continued to receive oral nutritional supplements as part of their ongoing nutritional management.	2
Schultz TJ, Kitson AL, Soenen S, Long L, Shanks A, Wiechula R, et al. Does a Multidisciplinary Nutritional Intervention Prevent Nutritional Decline in Hospital Patients? A Stepped Wedge Randomised Cluster Trial. e-SPEN Journal. 2014;9(2):e84-e90.	2014	3	1. Standard care (n=135)	1. Facilitator pairs consisting of a nurse from one of the step's wards and a dietitian were assigned to lead the nutritional intervention.<br>2. These facilitator pairs were responsible for educating and training staff on the use of the Malnutrition Universal Screening Tool (MUST), thereby enhancing the quality of nutritional assessments.<br>3. Additionally, they collaborated with the hospital kitchen to implement the use of red trays for specific nutritional needs, facilitating more tailored feeding assistance.	2
Sharan kumar VG, Pajanivel R, Boratne AV, Vimal Raj R. Impact of Dietary Counselling on the Nutritional Status and Quality of Life among Pulmonary Tuberculosis Patients - a Randomized Control Trial. Indian Journal of Tuberculosis. 2021.	2021	1	1. Patients were advised to take high protein diet, but the diet was not charted by the nutritionist.	1. A personalized dietary counselling approach was implemented, focusing on locally available and culturally acceptable foods that aligned with the patients' socio-economic status. This was facilitated through diet charts provided by the nutritionist.<br>2. Counselling was initiated at the beginning of each patient's treatment to ensure timely intervention and address any nutritional deficits or concerns.	2

Study	Year	Study Type (RCT =1, NRSI = 2, cRCT = 3)	Control	Intervention	Complexity of Intervention (Simple = 1, Complex = 2)
				3. Follow-up consultations were conducted via phone throughout the treatment duration, allowing for dynamic adjustments to the dietary plan as needed.	
Sharma Y, Thompson CH, Kaambwa B, Shahi R, Hakendorf P, Miller M. Investigation of the Benefits of Early Malnutrition Screening with Telehealth Follow up in Elderly Acute Medical Admissions. Qjm. 2017;110(10):639-47.	2017	1	1. Standard Care. Dietitian review occurs only if patients are referred by a health care professional. No dedicated outpatient follow-up after discharge. (n=46)	1. A multi-faceted nutritional intervention was deployed that included nutritional supplements, food fortification, and mid-meal snacks while patients were in the hospital. This was supplemented by dietary counselling provided by a dietitian to both patients and caregivers.<br>2. Ward-based staff were available to assist with meals as needed, and ongoing support was provided through monthly telephone calls from the dietitian for a duration of two months post-discharge. These calls aimed to reinforce adherence to the dietary regimen.<br>3. Compliance with the intervention was rigorously evaluated through a 24-hour self-reported dietary recall method.	2
Shyu YI, Liang J, Tseng MY, Li HJ, Wu CC, Cheng HS, et al. Comprehensive care improves health outcomes among elderly Taiwanese patients with hip fracture. J Gerontol A Biol Sci Med Sci 2013 Feb;68(2):188-97.	2013	1	1. The usual care group, consisting of 99 participants, received an average of 1.89 (SD = 2.32) physical therapy sessions during their hospitalization period.<br>2. Typically, these patients were discharged from the hospital approximately 5–7 days post-surgery. They received varied discharge health education from their primary nurses, but no organized home rehabilitation was provided.	1. The comprehensive care group received one year of in-home rehabilitation, including three in-home physical therapy (PT) sessions in the first four months following hospital discharge. These sessions were strategically scheduled within the first week, third week, and third month post-discharge. An additional PT session was administered at the sixth-month mark.<br>2. Beyond the rehabilitation framework, the comprehensive care regimen also incorporated other holistic elements such as geriatric consultation, discharge planning with post-hospital services, and a structured rehabilitation program.<br>3. Additional facets of the comprehensive care model included specialized consultations in nutrition, depression management, and fall prevention, offering a multi-disciplinary approach to patient care	2

Study	Year	Study Type (RCT =1, NRSI = 2, cRCT = 3)	Control	Intervention	Complexity of Intervention (Simple = 1, Complex = 2)
			3. Post-discharge, the usual care group underwent clinical follow-ups at predetermined time points—specifically, at 1, 3, and 6 months—to assess their recovery and health status.		
Singh NA, Quine S, Clemson LM, et al. Effects of high-intensity progressive resistance training and targeted multidisciplinary treatment of frailty on mortality and nursing home admissions after hip fracture: a randomized controlled trial. J Am Med Dir Assoc 2012;13:24–30.	2012	1	1. Standard care as offered for hip fracture in the area health service, including orthogeriatric care, rehabilitation service, other medical and allied health consultation as required, and physiotherapy.	1. The experimental group underwent high-intensity resistance training at 80% peak strength, supervised bi-weekly for 12 months in an outpatient clinic.<br>2. Training started 6-8 weeks post-fracture, following standard physiotherapy. Coordination was managed through weekly team meetings.<br>3. Participants received monthly home visits and phone calls from trainers, totalling 80 training sessions, 10 visits, and 10 calls over a year.	2
Söderström L, Rosenblad A, Bergkvist L, Frid H, Thors Adolfsson E. Dietary Advice and Oral Nutritional Supplements Do Not Increase Survival in Older Malnourished Adults: A Multicentre Randomised Controlled Trial. Upsala Journal of Medical Sciences. 2020;125(3):240-9.	2020	1	1. Standard Care	1. Dietary Counselling Group: Patients received a single counselling session by a dietitian before discharge, with no supplements. Follow-ups at 1, 3, and 6 months via phone.<br>2. Supplement Group: Patients were instructed to consume 1–2 supplement bottles daily. 100% compliance. Follow-ups at the same intervals.<br>3. Combined Group: Received both dietary counselling and 1-2 daily supplement bottles. Follow-up calls at 1, 3, and 6 months post-discharge.	2
Starke J, et al. Short-term individual nutritional care as part of routine clinical setting improves outcome and quality of life in malnourished medical patients. Clin Nutr 2011;30(2):194-201.	2011	1	1. Standard nutritional care, including the prescription of ONS and nutritional therapy prescribed by the physician	1. Comprehensive Nutritional Assessment: Participants received an individualized assessment to tailor their food supply.<br>2. Meal Fortification: Regular meals were enhanced with maltodextrin, rapeseed oil, cream, and/or protein powder to meet nutritional needs.	2

Study	Year	Study Type (RCT =1, NRSI = 2, cRCT = 3)	Control	Intervention	Complexity of Intervention (Simple = 1, Complex = 2)
			independently according to the routine ward management.	3. Additional Nutritional Support: In-between snacks and oral nutritional supplements were provided to supplement dietary intake.	
Stelten S, Dekker IM, Ronday EM et al. (2015) Protein enriched "regular products" and their effect on protein intake in acute hospitalized older adults; a randomized controlled trial. Clin Nutr 34, 409–414	2015	1	1. Regular bread and regular drinking yoghurt ad libitum.	1. Protein-enriched bread (Carezzo bv®) and protein-enriched drinking yoghurt (Drinking yoghurt enriched with Fonterra Whey Protein Concentrate (WPC. 515) by Fonterra Europe®) ad libitum.	1
Terp R, Jacobsen KO, Kannegaard P, Larsen AM, Madsen OR, Noiesen E. A Nutritional Intervention Program Improves the Nutritional Status of Geriatric Patients at Nutritional Risk—a Randomized Controlled Trial. Clinical Rehabilitation. 2018;32(7):930-41.	2018	1	1. Standard Care. The clinical dietician was involved in the process if the patient had specific needs and gave dietary advices and prepared a dietary plan for nutrition intake while they were hospitalized.<br>2. At discharge, no follow-up was planned.	1. Individualized Plans: Dietitian-crafted plans based on everyday food, potentially supplemented.<br>2. Post-Discharge Care: Dietary advice for after hospital release.<br>3. Follow-Up: Three visits at 1, 4, and 8 weeks post-discharge, conducted by district nurse or healthcare assistant.	2
Van den Berg GH, Lindeboom R, Van der Zwet WC. The effects of the administration of oral nutritional supplementation with medication rounds on the achievement of nutritional goals: a randomised controlled trial. Clinical Nutrition 2015; 34:15-19.	2015	1	1. Usual care group was offered 125 ml of ONS by nutrition assistants twice a day in between meals at 10 and 15 o'clock. (n=88)	1. Group I: 125 ml ONS twice daily at 12 and 17 o'clock, aligned with medication.<br>1. Group II: 63 ml ONS four times daily at 8, 12, 17, and 20 o'clock, also with medication.<br>2. Participant Count: Both interventions involved 146 subjects.	1
Vázquez-Sánchez M, Valero-Cantero I, Carrión-Velasco Y, Castro-López P, Suárez-Cadenas E, Casals C. Applicability and Clinical Validity of Nursing Outcomes Classification in a Nursing Intervention of Nutritional Counseling for Patients with Malnutrition. Int J Nurs Knowl. 2019;30(3):168-72.	2019	1	1. Standard care	1. Patients underwent nutritional counselling by case manager nurses, which began during the hospital stay and lasted for six months.	1

Study	Year	Study Type (RCT =1, NRSI = 2, cRCT = 3)	Control	Intervention	Complexity of Intervention (Simple = 1, Complex = 2)
Vermeeren MA, et al. Nutritional support in patients with chronic obstructive pulmonary disease during hospitalization for an acute exacerbation; a randomized controlled feasibility trial. Clin Nutr 2004;23(5): 1184-92.	2004	1	1. Placebo ONS	1. ONS 125 ml x 3 serves/d (Respifors, Nutricia, Zoetermeer, The Netherlands, 2.38 MJ/day, 20 energy% protein, 20 energy% fat and 60 energy% carbohydrate)	1
Vlaming S, et al. Should the food intake of patients admitted to acute hospital services be routinely Supplemented? A randomized placebo controlled trial. Clin Nutr 2001;20(6):517-26.	2001	1	1. Placebo ONS	1. ONS 400 ml/d (Ensure Plus, Abbott Laboratories Ltd)	1
Volkert D, et al. Nutritional support and functional status in undernourished geriatric patients during hospitalization and 6-month follow-up. Aging Clin Exp Res 1996;8(6):386-95.	1996	1	1. Standard hospital diet	1. In addition to the standard hospital diet, patients were offered 2 portions of a liquid nutritional supplement (200 mL soup in the midmorning, 200 mL sweet drink in the after- noon) daily.<br>2. Different brands with similar composition but different flavours were used in order to increase variety and acceptance	2
Walton K, Williams P, Bracks J, Zhang Q, Pond L, Smoothy R, et al. A volunteer feeding assistance program can improve dietary intakes of elderly patients - a Pilot Study. Appetite 2008;51(2):244-8.	2008	2	NA	1. 25 volunteers, specifically recruited and trained (time unspecified)<br>2. 45 min to assist two to three patients with their lunch meal.	2
Wong A, Burford S, Wyles CL, Mundy H, Sainsbury R. Evaluation of strategies to improve nutrition in people with dementia in an assessment unit. J Nutr Health Aging 2008;12(5):309-12	2008	2	NA	1. Providing a glass door refrigerator filled with snacks and beverages to which patients have access 24 hr a day, seven days a week.<br>2. Playing soothing music at meal times	1
Wong SY, Lau EM, Lau WW, Lynn HS. Is dietary counselling effective in increasing dietary calcium, protein and energy intakes in patients with osteoporotic fractures? A randomised controlled clinical trial. Journal of Human Nutrition & Dietetics 2004;17(4):359-6	2004	1	1. 500 mg of calcium supplementation and an anti-resorptive agent for the treatment of osteoporosis.<br>2. Given a printed pamphlet, on the	1. 500 mg of calcium supplementation and an anti-resorptive agent for the treatment of osteoporosis.<br>2. A one to one 45 minute session with a dietitian. Each patient received education about the importance of adequate calcium and protein in their diet.	2

Study	Year	Study Type (RCT =1, NRSI = 2, cRCT = 3)	Control	Intervention	Complexity of Intervention (Simple = 1, Complex = 2)
			prevention of osteoporosis.		
Wright L, Cotter D, Hickson M. The effectiveness of targeted feeding assistance to improve the nutritional intake of elderly dysphagic patients in hospital. J Hum Nutr Diet 2008;21(6):555-62.	2008	2	NA	1. 1-week training programme by an experienced Dietitian and SALT on targeted assistance	2
Wyers CE, Reijven PL, Breedveld-Peters JJ, Denissen KF,Schotanus MG, van Dongen MC, et al. Efficacy of nutritional intervention in elderly after hip fracture: a multicenter randomized controlled trial. Journals of Gerontology. Series A,Biological Sciences and Medical Sciences 2018;73(10):1429-37.	2018	1	1. Standard care	1. Regular counselling by a trained study dietitian<br>2. ONS for 3 months post-surgery, started during hospitalization and continuing in the rehabilitation centre and/or, if applicable, at home.<br>3. Throughout the study, a patient was supervised by the same dietitian. Dietary advice, based on individual energy and protein requirements, comprised an energy- and protein-rich diet as well as recommendations on choice, quantity, and timing of foods. (400 mL per day) for 3 months.<br>4. The dietitian had ten contacts with each patient: two during hospitalization and eight thereafter (three face-to-face and five telephone calls).	2
Yang PH, et al. Effect of nutritional intervention programs on nutritional status and readmission rate in malnourished older adults with pneumonia: a randomized control trial. Int J Environ Res Publ Health 2019;16(23).	2019	1	1. Standard ONS according to the Kaohsiung Chang Gung Memorial Hospital Nutrition Department	1. Individualized nutrition plan crafted by a dietitian, involving both patients and their caregivers. The post-discharge diet was taught with dietary advice.<br>2. Unspecified percentage received supplements as part of their dietary plan.<br>3. Post-discharge follow-up included phone calls to monitor nutritional status and adapt plans; the frequency of calls was not reported.	2
Yoshimura Y, Uchida K, Jeong S, Yamaga M. Effects of ONS on Muscle Mass and Activities of Daily Living in Elderly Rehabilitation Patients with Decreased Muscle Mass: A Randomized Controlled Trial. J Nutr Health Aging. 2016 Feb;20(2):185-91.	2016	1	1. Resistance training only	1. ONS (Resource PemPal Active 200kcal/10g protein) + resistance training	1

Study	Year	Study Type (RCT =1, NRSI = 2, cRCT = 3)	Control	Intervention	Complexity of Intervention (Simple = 1, Complex = 2)
Young AM, Banks MD, Mudge AM. Improving nutrition care and intake for older hospital patients through system-level dietary and mealtime interventions. Clin Nutr ESPEN. 2018;24: 140-147.	2018	2	NA	1. Added 1.0 FTE assistant-in-nursing (AIN) to help with meal set-up, feeding, and encouragement during meals and snacks.<br>2. Quarterly multidisciplinary education sessions aimed at junior medical officers and allied health professionals, although the specifics of the training were not detailed.<br>3. An additional 0.5 FTE multi-professional allied health assistant (AHA) was implemented to handle tasks to mitigate functional decline, delirium, and malnutrition, including meal assistance. No other changes to staffing levels.	2
Young, A.M., Mudge, A.M., Banks, M.D., Ross, L.J., Daniels, L., 2013. Encouraging, assisting and time to EAT: Improved nutritional intake for older medical patients receiving protected mealtimes and/or additional nursing feeding assistance. Clin. Nutr. 32, 543–549. doi:http://dx.doi.org/10.1016/j.clnu.2012.11.009	2013	2	NA	1. Whole-team approach with no staffing changes, but strategies were negotiated to prioritize mealtimes by minimizing non-urgent tasks and scheduling interruptions outside of mealtimes.<br>2. Comprehensive 25-hour clinician education program, inclusive of 6 hours for nursing, aimed at reinforcing the importance of patient nutritional intake.<br>3. Addition of one assistant-in-nursing staff per ward to assist registered nurses and dietitians, specifically focused on monitoring plate waste and providing early nutrition support to "at-risk" patients.	2
